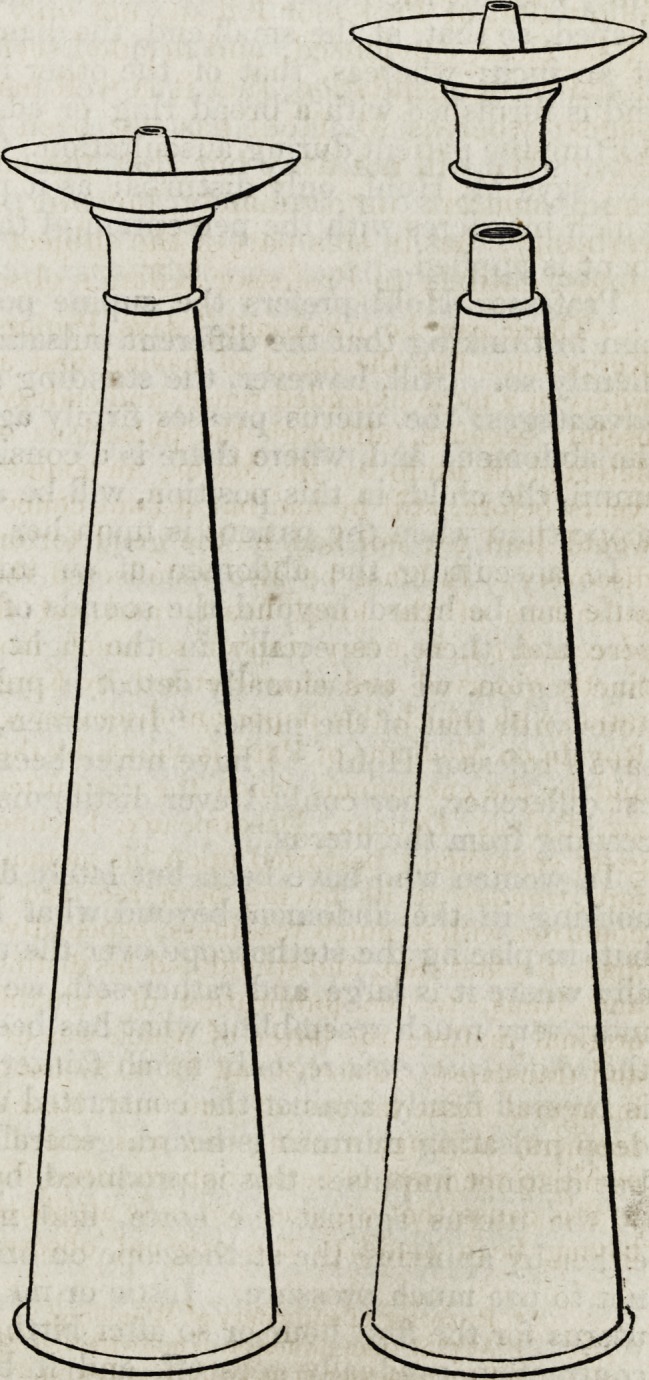# Obstetric Exploration

**Published:** 1836-01

**Authors:** 


### Art. IV.

*Die geburtshülfliche Exploration, von* Dr. Anton Friedrich Hohl, ausserordentlichem Professor der Universität zu Halle, u. s. w. *Erster Theil: Das Hören.—Halle,* in Januar, 1833. 8. 314 s; *mit einem Kupfertafel. Zweiter Theil: Das explorative Sehen und Fühlen, nebst einem Anhange.—Halle,* in Januar, 1834. 8. 438 s.

*Operationslehre für Geburtshelfer, in zwei Theilen.* Von Dr. Hermann Friedrich Kilian, ordentl. öffentl. Professor der Geburtshülfe und der Geburtshülflichen Klinik an der Rheinischen Friedrichs-Wilhelms Universität, u. s. w. *Erster Theil: die Operative Geburtshülfe.—Bonn,* 1834. 8. 478 s.

*Obstetric Exploration;* by Anthony Frederic Hohl, m.d., Professor Extraordinary at the University of Halle, &c. *Part First: Auscultation.—Halle,* 1833. 8vo. pp. 314; *with an Engraving. Part Second: Exploratio per Visum et Tactum; with an Appendix.—Halle,* 1834. 8vo. pp. 438.

*Instructions in Operating for Accoucheurs, in two Parts.* By Herman Frederic Kilian, m.d., Public Professor in ordinary of Midwifery, and of the Midwifery Clinic at the University of Bonn, &c. *Part First: Operative Midwifery.—Bonn.* 8vo. pp. 478.

The subject of obstetric diagnosis is only beginning to receive that degree of attention which its great importance demands. During the last few years, either from the increased facilities of communication with the continent, or from the general advance of science, and anxiety to investigate truth and lay aside the all-obscuring veil of theory, accoucheurs in this country have evinced a laudable zeal for its improvement: several valuable works on obstetrics have been published, and a variety of excellent memoirs have appeared in our Medical Journals. Much interest has thus been excited to the labours of our continental brethren, and most of the French obstetric works of the present day are in the hands of the English accoucheurs. Those of Germany have not been so much favoured; and it shall be our care not only to lay before our readers such works from that country as will acquaint them with the present state of knowledge there, but also to give such a selection and digest of that knowledge as may render it available and useful. The labours of our German brethren in the department of midwifery demand our attention and respect: their profound learning, patient investigation, unimpeachable honesty, methodical arrangement, and indefatigable industry, render them peculiarly valuable. The verbose diffuseness, of which they have been so long (and perhaps justly) accused, is beginning to disappear: few of the really valuable German writers are guilty of this fault; and by this criterion alone we may often form a tolerably correct opinion as to the merits of a work. We do not, however, intend to let this be our guide in reviewing the two works before us, but shall carefully investigate their merits, selecting whatever we think will prove of interest or utility.

We have combined the first chapter of Professor Kilian's work on Operative Midwifery with that of M. Hohl, since they treat upon the same subjects, with the only difference (and it is no slight one,) that what the former discusses in ninety-seven pages, the other dilates into two tolerably-sized octavo volumes.

Professor Hohl has devoted his first volume to the consideration of obstetric auscultation; a subject of increasing interest and importance, to which he appears to have devoted much attention and labour. Both works have given a view of its history, and we shall follow that of Professor Kilian, not only from being recommended by its clear, condensed, and decided style, but also from the additional recommendation, that it is well handled in the course of two pages; whereas M. Hohl has not been able to do with less than fifty. We will not deny but that the latter has given some interesting matter: unfortunately, however, it is so diluted by prolix verbosity, that in this part of the subject we must confine ourselves almost entirely to Professor Kilian's observations.

"There can be no doubt," says Professor K., "that Lejumeau de Kergaradec has the merit of having first applied auscultation to midwifery in a more extended sense, and endeavoured to make it practically useful; because, although the pulsations of the fœtal heart had been detected in utero by M. Major, an eminent surgeon of Geneva, two years before, still he had but a faint conception of the results which it would lead to; nor had he the good fortune to take advantage of his discovery. It must be also recollected that Major detected only the pulsations of the fœtal heart, and not the placental sound *(souffle placentaire)*, in every respect so much more important, and first distinctly observed and characterized by Kergaradec. The high consideration with which this important discovery was received by the Académie Royale de Médicine of Paris is shown by their Transactions at that time, and by the encomiums which the committee who had to report upon it, viz. Dubois, Deneux, Desormeaux, Laennec, and De Lens, (names of great eminence,) bestowed upon his memoir. We can scarcely, therefore, wonder at its having been received by eminent men of different countries with so much readiness, especially in Germany. D'Outrepont* made the first step, and stimulated his distinguished pupils, Drs. Ulsamer and Haus, to the publication of valuable essays, containing much original matter; the latter of which was even acknowledged in France, and translated. Busch† and El. v. Siebold‡ were not inactive, and the inaugural dissertations of Lau and Reccius evince the zeal of their teachers, although nothing new was added to what was already known, owing to the want of better opportunities for making observations. It is to this cause that we must not only attribute the fact of so little addition having been made to the original observations of Kergaradec for a

* Professor of midwifery at Würzburg.

† At that time professor of midwifery at Marburg, now at Berlin.

‡ Late professor of midwifery at Berlin.

number of years, but that this important discovery began to be almost forgotten, or looked upon with indifference and doubt. The last few years, in this respect, have made amends for the neglect of the previous ones: the original views of Kergaradec have been carried on, and improved upon, most successfully; having attained a much greater degree of certainty, and much more practical value. The labours of Schottin, D'Outrepont, Kennedy, and Paul Dubois, deserve especial mention: among them, those of Kennedy rank very high, although we look upon the classical memoir of P. Dubois as decidedly the most complete, and by far the best upon the subject; he having, with astonishing patience, accuracy, and talent, ausculted no less than three hundred pregnant and parturient women, and obtained the most interesting results."

Dr. Hohl agrees with most other obstetric auscultators, that the *mediate* auscultation is far preferable to the *immediate;* or, in other words, that it is much better to apply the stethoscope than merely to use the ear. In this we fully coincide with him: the sounds are heard clearer, more distinct, and less mixed with the murmur of the arteries in the immediate vicinity of the ear itself. Dr. Hohl has given a description and drawing of the stethoscope he is in the habit of using. We will translate the one, and give a copy of the other; because the instrument seems, on the whole, constructed on philosophical principles, and because we are anxious that all alleged improvements, in so important a department of practical medicine, should be made as widely known as possible. We have ourselves, as yet, had no practical experience of the instrument; and we confess we are apprehensive that the projecting nipple on the auricular extremity will be found rather a disadvantage than an advantage.

The instrument consists of two pieces, viz. the ear-piece and tube. The former is a concave disc, two inches in diameter: its depth, which gradually increases towards the centre, is at this point about one sixth of an inch. In the middle there is a small conical tube, or nipple, a quarter of an inch long, to correspond with the external auditory passage, and thus prevent the opening being closed by the pressure of the disc, which, from its concave form, is well adapted to the external ear. On the convex surface of this disc is a neck, into which the extremity of the tube fits. The tube, without the neck, is nine and a half inches long, and funnel-shaped, so that, at the small end, the diameter is only three eighths of an inch; whereas, that of the other is two inches. The large end is furnished with a broad ring or edge, in order to prevent its hurting the patient during auscultation. Closing the [unemployed] ear, says M. Hohl, only disturbs; as it produces a boiling sound, which interferes with the perception of the ear to which the instrument is applied.

Professor Hohl prefers the supine posture, and we agree with him in thinking that the different pulsations are heard most conveniently so. Still, however, the standing position has its occasional advantages: the uterus presses firmly against the anterior wall of the abdomen, and, where there is a considerable quantity of liquor amnii, the child, in this position, will be actually nearer the stethoscope than when the patient is upon her back.

In ausculting the abdomen of an unimpregnated woman, but little can be heard beyond the sounds of the gas in the intestines: here and there, especially in the right hypochondrium and left iliac region, we occasionally detect a pulsating murmur, synchronous with that of the pulse. "In women, at the menstrual period," says Professor Hohl, "I have never been able to detect the slightest difference, nor could I ever distinguish the smallest sound proceeding from the uterus."

In women who have been but lately delivered, we hear little or nothing in the abdomen beyond what has been just mentioned; but, in placing the stethoscope over the uterus at this time, especially where it is large and rather soft, we shall hear a distant murmur, very much resembling what has been described by authors as the *souffle placentaire,* only much fainter. When the stethoscope is pressed firmly against the contracted uterus, directly in front, a deep pulsating murmur is heard, generally accompanied by a slight but distinct impulse: this is produced by pressing the solid mass of the uterus against the aorta, and may be generally avoided, either by applying the stethoscope on one side, or by being careful not to use much pressure. Little or no sound will be heard in the uterus for the first hour or so after birth; but, as the state of firm contraction gradually goes off, and it becomes larger and softer, and the blood again fills its spongy parietes, we now begin to perceive a low gentle souffle, like what we heard during the gravid state. M. Hohl observes, that this is increased during sharp after-pains, and that, in from five to seven days after birth, when the uterus has become hard and contracted, all trace of this sound is lost; but we are surprised that he makes no mention of the sound which is accompanied with impulse, and which is evidently produced by the aorta behind the uterus, and which may be heard certainly for seven, if not more, days after labour.

In availing ourselves of the account of auscultation during pregnancy, given by our authors, we must in great measure follow (as far as he goes,) Professor Kilian, on account of the clear and concise manner with which the descriptions are given.

"On ausculting the different parts of an abdomen which is distended by the gravid uterus, two species of pulsation will be distinguished: they are essentially different from each other, and are heard at different parts of the abdomen. In the first place, we shall hear, about the middle of the gravid uterus, a little to one side, and generally the left, distinct double pulsations, which follow each other very rapidly, and are mostly, although not always, in perfect rhythm; and, secondly, in various parts of the uterus, but most distinctly towards the fundus, and especially on the left, we shall also perceive very audible single pulsations, accompanied with a peculiar tone or murmur. The first sound is from the *pulsations of the fœtal heart;* the second is from the *murmur of the circulation through the gravid uterus.*"

We will follow the observations of the Professor at Bonn, where he enters more minutely into the description of these two sounds.

"*Pulsation of the Fœtal Heart.* In the first place, we must notice a fact, which we might almost expect, viz. that these pulsations, as well as the sound of the circulation, are heard much more distinctly after the discharge of the liquor amnii than before. Still the presence of the liquor amnii is never a hinderance of any importance, if the stethoscope be placed on the right spot; but it may easily happen that, during auscultation, either from the movements of the child or the patient herself, sometimes more, sometimes less liquor amnii is interposed between the fœtus and wall of the uterus; so that at one moment the fœtal pulsations will be heard stronger, at another weaker, and occasionally so weak as to be scarcely distinguishable. In order to avoid these varieties, and hear the sound as distinctly as possible, the best way is to press the stethoscope deep into the abdominal parietes, and thus bring it as near as possible to the child's body. These facts, which we have frequently observed in our own examinations, we are glad to find confirmed by P. Dubois.

"We usually hear from 140 to 150 double beats in a minute; nor does the age of the fœtus appear to have any influence upon the number of beats, because, about the time when the movements of the child can be just perceived, viz. between the eighteenth and twentieth week, and when the fœtal circulation can be first certainly heard, although with great difficulty, their number is precisely the same as above mentioned.

Yet sometimes, although only for a few moments, all at once, and without any assignable reason, (since nothing which accelerates the pulse of the mother affects that of the child,) the number of beats increases very considerably; diminishing at the same time so remarkably in power as to be almost inaudible. In ascertaining the situation of these pulsations, whether it be late or early in pregnancy, we find that they are not confined to a very small extent, but may be heard over a space of four fingers' breadth, and sometimes even more: the extent over which they can be heard is less when but a small quantity of liquor amnii is present, or where it has escaped: here, what we lose in extent, we gain in intensity of sound."

On the other hand, as M. Hohl observes, "it will occasionally happen that, for whole hours, the double pulsations will be only 106 or 108 in a minute; whereas, both before and after, it ranged from 140 to 154."

"2dly. *Sound of the Circulation in the Gravid Uterus.* This is the sound which has been characterized by the name of *souffle placentaire* by Kergaradec, and by the German accoucheurs by that of *Placentargeräusch,* (placental murmur.) It is much more easy to detect by auscultation than the other, and especially by the ear alone. It was all very excusable to suppose that this sound, which is chiefly perceived towards the fundus uteri, and frequently at the spot where the placenta is usually attached, was produced by the active and sonorous circulation of this vascular mass; and, until very lately, no peculiar objections have been raised against it. Haus, it is true, expressed a suspicion that this sound might be produced by the aorta and iliac arteries, but did not venture to give a decided opinion; and Ulsamer, who has given some really excellent remarks, and who came very near the truth, could not bring himself to differ from the authority of the French observers, but has retained the erroneous name of placental pulsation. The accurate and successful investigations of Schottin and D'Outrepont, and especially those of Kennedy and Dubois, with which our own observations fully agree, first proved beyond all doubt that the sound in question did not point out the situation of the placenta, but that it depended upon the vascularity and arrangement of vessels peculiar to the gravid uterus, and that it was exclusively produced by this. This sound is only to be heard in the gravid uterus, and may be perceived in most parts of its anterior wall: it is most distinct at those spots where the vessels have undergone the greatest increase of size and degree of contortion, viz. about the fundus, and especially at its sides. We shall not venture to determine which side is most favourable for auscultation; but of this we are perfectly certain, that it is very far from being always on that side at which the placenta is afterwards found attached. This circumstance, as also the fact that the above sound may be sometimes heard in directly opposite parts of the uterus, sometimes over the whole extent of its anterior wall, and even at the lowest part of its inferior segment, (a fact we have frequently observed in multiparæ,) and also, of which there can be now no doubt, the pulsations having been heard in cases where it was ascertained that the child was dead, and even in women shortly after labour, (Kennedy and Dubois) prove unequivocally that the placenta has nothing to do with these '*battemens avec souffle*,' as they have been termed by the French."

Professor Hohl follows the views of the French authors, and attributes the above-mentioned sound to the circulation in the placenta, or, at least, in that part of the uterus where the placenta is attached. Whatever Dr. Kennedy's views may have been, they have been considerably modified in his "Observations on Obstetric Auscultation:" he says, (p. 69,) "upon the transmission of the blood through the arteries of that part of the uterus to which the placenta is attached, the phenomenon in question, the souffle, principally depends:—we say *principally*, because it appears that it may also be produced by the passage of the blood at the lateral part of the uterus, above alluded to, without the placenta being attached directly to that part:" and, in the following page (70), he says, "The placental sound is present in pregnant women only while the uterine circulation is connected with that of the placenta, and ceases when the vessels which serve to sustain this connexion are no longer pervious; a fact which we can ascertain by examining a woman shortly before parturition, when we may observe this phenomenon in full energy; and again when the uterus is empty and perfectly contracted after delivery, or when the fœtus, having died *in utero*, a complete obstruction in this system of vessels is produced; in which cases, not the slightest vestige of the phenomena can be discovered." An apparent contradiction occurs a few lines further on, respecting which we have never been able perfectly to satisfy ourselves. "It is always heard either in that part of the uterus to which the placenta is or *has been* attached." Again, at p. 222, he says, "Cases occasionally occur, however, in which the utero-placental circulation appears to be kept up very freely for some time after the death of the fœtus; and, whilst this is the case, the souffle neither ceases, nor is it observed so completely altered in its character." Dr. Kennedy does allow that, after labour, where the uterus is not firmly contracted, and soft enough to admit of blood circulating through its structure, a distinct souffle will be perceived. With this view we fully agree, and, from observations which we made some years ago relative to the size of the uterus at different periods after labour, we can easily account for the souffle heard at this period. The pulsation which is communicated to the uterus after labour has not escaped Dr. K.'s notice.

This murmur of the uterine circulation may be heard quite as soon as, or even at an earlier period, than the pulsations of the fœtal heart; "at least," as Professor Kilian observes, "we can often hear the one quite distinctly when the other is scarcely perceptible." Professor Hohl has never been able to detect the sound of the uterine circulation before the fourth month; but Dr. Kennedy has given some very interesting cases, where he was able to hear it with certainty at the twelfth, eleventh, and even in one case possibly at the tenth, week. We should be inclined to use this fact as a further corroboration of Professor Kilian's views. At this early period it is well known that the chorion is almost entirely covered with the tufted venous radicles which form the future placenta: they are beginning, it is true, to disappear from the lower part of the ovum, but still by far the greater portion is covered with them: in fact, there is no placenta at present; the placental decidua does not yet exist; the peculiar placental circulation is not yet established: the souffle, therefore, here can only be produced by the circulation in the parietes of the uterus; and yet none of its vessels have attained any thing like the size, or display those intricate contortions, which are observed at a later period. Surely, if a distinct sound is produced at this early period of uterine development, we are justified, *a priori*, in expecting to find the souffle at every point of the uterus within reach of our stethoscope, at a later period. Our own observations have for some time unequivocally led to this conclusion, and we could have wished—and we say it with every feeling of respect,—that so experienced and accurate an observer as Dr. Kennedy had come to a more decided result.

With regard to the period at which the pulsations of the fœtal heart can be first heard, all agree that they can seldom be detected before the beginning of the fifth month, and are then so faint as to require great attention and patience. M. Hohl has devoted a long and wearisome chapter to the relation between the fœtal pulsations and the mother's pulse, and has, in our opinion, wasted a great deal of time in instituting a series of observations to prove that the fœtal pulsations are entirely independent of the mother's pulse. After having examined the two pulses with the patient supine, sitting, or standing, he actually auscults her when asleep, and gravely laments that he had not an opportunity of doing it while she was dreaming. He thinks that the temperature of the mother has some effect on the fœtal heart, but so little as to be very uncertain. Passions and affections of the mind, spirituous liquors, &c. have no effect on the fœtal heart. In cases of small-pox, the fœtal pulsations continued unaffected, but, where petechiæ appeared, a change was soon perceptible, and they quickly became inaudible. In a very severe case of hæmorrhagia petechialis, with bleeding from the gums and bloody urine, M. Hohl heard the fœtal pulse distinctly on the 8th of the month: the woman sickened during the night, and, on the following day, the appearance of the skin already showed the character of the disease. He had distinctly heard the double pulse early that morning, although weaker, but by noon it had disappeared: no sound was to be heard throughout the whole uterus. The patient died on the 12th. In mild cases of Asiatic cholera, no change was observable in the fœtal pulse; but, where the prostration and anxiety were very great, and the pulse at the wrist imperceptible, the sound of the fœtal heart became much quicker, and soon stopped.

Professor Hohl has had the opportunity of ausculting two cases where menstruation continued to appear during pregnancy: in one case it ceased at the end of the fifth month, in the other at the end of the seventh month: in both the discharge was moderate, but perfectly regular in its appearance.

"With regard to the fœtal pulse," says M. Hohl, "no change was observable; which is the more remarkable, as a considerable excitement appeared throughout the whole vascular system of the patient. The souffle not only participated in this periodic exacerbation, but presented also a remarkable phenomenon, which I have only observed in labour during the presence of a pain, viz. that the tone of the pulsations rose periodically above the usual pitch, and was accompanied with a peculiar strong piping sound, as if from separate vessels: the intensity of this sound appeared to me to be in direct proportion with the increased or diminished congestion of the uterus, and seems to mark the commencement of labour, which usually comes on at what would have been a menstrual period."

During labour, a considerable variety is heard in the tone, locality, and number of the pulsations, especially of the uterine circulation. This latter becomes stronger and more distinct, and remarkably variable in its tones, just before a pain comes on: even if the patient were inclined to conceal her pains, this peculiar change, and more especially the rapidity of the beats, would enable us to ascertain the truth. "The moment a pain begins," as M. Hohl observes, "and even before the patient herself is aware of it, we hear a sudden short rushing sound, which appears to proceed from the liquor amnii, and to be partly produced by the movement of the child, which seems to anticipate the coming on of the contraction. Almost at the same instant all the tones of the uterine circulation increase in intensity; other tones, which had not before been heard, and which are of a piping resonant character, now become audible, and seem to sound through the tube of the instrument, just like a string which has been twanged, and drawn tighter whilst vibrating."

This alteration in the tone of the uterine circulation is very remarkable, and it has more than once puzzled us to account for it: we had been in the habit of comparing it with the sound which a stone produces when thrown obliquely so as to fly in repeated bounds over an extensive sheet of ice; each time that it strikes the ice, it produces a short sharp twanging sound, exactly similar to that of the uterine circulation at this time. M. Hohl's comparison, however, gives an excellent idea of it. Dr. Kennedy, also, in one or two cases, has observed a metallic resonance in the pulsations of the fœtal heart, but cannot account for the cause of it. To return to M. Hohl's observations.

"As soon, however, as the pain has reached its summum of intensity, and again begins gradually to disappear, the murmuring character of the pulsations (uterine) returns as gradually, sounds as full as at the beginning of the pain, and has now its former tone as before labour, only somewhat louder. These phenomena occur if the pain be a genuine labour-pain, and runs its course regularly; but, in spurious or irregular pains, it is quite different: the sound remains unaltered, or increases only for an instant."

Neither the increase of the pulse nor temperature of the body, which are observed during labour, have any effect upon the fœtal heart. During every pain, the pulse and uterine murmur undergo a slight but regular series of changes; as the pain increases, the rapidity of the pulse increases also, it attains its height with the pain, and again diminishes with it, and continues at its former rate until the pain returns. The more regular the pains are, and the more gradually they rise to their height and again diminish, the more distinctly will this phenomenon be observed. "We may," as Prof. Hohl observes, "reverse it; the more distinctly the increase of sound betokens the approach of a pain, and the more regularly the beats increase in rapidity, and the more decidedly they reach a certain intensity, remain there for a little, and again gradually diminish, the more genuine will the pain be, the more completely will it reach its full power, and the more efficacious will be its action on the progress of labour." M. H. has collected, with great industry, a considerable number of observations to prove this point, the results of which are very interesting: they are, it is true, needlessly diffuse, but this fault is less remarkable wherever the matter is so good.

We must, however, pass over another of those deserts which the reader occasionally meets with in Professor Hohl's work: a few further observations are given as to the relation between the pulse and course of the pains, which are interesting, and an ingenious tabular form is given to show this connexion.

Our author considers that the *souffle* is only heard at that spot of the uterus where the placenta is attached, and gives sundry reasons for his being of this opinion. He says that we shall hear the souffle over the spot where the placenta is attached, which we admit; but when he says that the souffle does not extend beyond the circumference of the placenta, and is not heard elsewhere, this we must crave permission to deny: our reasons for saying so have been already stated. He has devoted an equally long chapter to prove that the double pulsations arise from the fœtal heart; and, although this prolixity is both wearisome and needless, he has interspersed several very interesting and valuable cases, especially two cases of triplets, in the latter of which the patient was ausculted carefully during labour. He agrees with Dr. Haus in saying that it is impossible to hear the pulsations of the cord in the uterus: the observations however of Dr. Kennedy, which were published during the same year (1833), and which have every appearance of being correct, lead to a very different result.

"In some cases," says Dr. Kennedy, (p. 121,) "where the uterus and parietes of the abdomen were extremely thin, I have been able to distinguish the funis by the touch externally, and felt it rolling distinctly under my finger; and then, on applying the stethoscope, its pulsations have been discoverable, remarkably strong; and, on making pressure with the finger for a moment on that part of the funis which passed towards the umbilicus of the child, I have been able to render the pulsation less and less distinct, and even, on making the pressure sufficiently strong, to check it altogether."—"The funis exhibits another phenomenon, which, on my first paying attention to obstetric auscultation, embarrassed me a good deal to find a rational explanation of,—namely, a *souffle*, which is occasionally met with, distinct from that of the placenta, and differing from it in character as well as situation. It is heard generally at a point of the uterus quite distinct from the placental souffle, is weaker and shorter in its duration, wanting in great measure the very protracted sibilous or hissing sound existing in so marked a degree in the other, and corresponding in frequency, not with the pulse at the wrist of the mother, but with the fœtal pulsation or the action of the child's heart."*

The eighth and last chapter of Professor Hohl's first volume is devoted to the application of auscultation to practical midwifery: it commences with it as a means of diagnosis in pregnancy. We should have supposed that this subject had been already sufficiently discussed; not so however in M. Hohl's opinion: every symptom which has ever been considered as a symptom of pregnancy is dwelt upon as if the author were afraid we should arrive at the end of the chapter too soon. He has given two or three interesting cases of doubtful pregnancy, where the usual changes in the form of the os uteri had been remarkably deceptive, and where auscultation had shown the real nature of the case. On the diagnosis of plural births, the stethoscope offers but little assistance, in comparison to what it does in other cases. If two different sets of fœtal pulsations can be heard, no one, of course, in his senses, would doubt the presence of more than one child; but we cannot always expect that circumstances will be so favourable as to enable us to hear both at the same moment. One child may be so situated as to be out of reach of the stethoscope, or it may be dead. The

* The reader will observe, that, in the foregoing observations respecting the cause of the *souffle* accompanying pregnancy, we have taken no notice of the recent opinion of M. Bouillaud, that it is attributable to the compression of some one or more of the large vessels of the abdomen, as the hypogastric and external iliac arteries, by the uterus charged with the product of conception. (*Traité clinique des Maladies du Cœur; Paris,* 1835.) Our reasons for this omission are, first, that our observations were written before we had an opportunity of seeing M. Bouillaud's work; secondly, because the subject is not noticed by the authors of the works under review; and, lastly, because there will be an opportunity, either in the present or next Number of this Journal, of more particularly considering this point, when noticing the recent works of MM. Bouillaud and Raciborski. We would, at present, only observe, that we do not consider the question as completely set at rest, and reserve to ourselves the right of changing our opinion, on the production of evidence which shall appear to us more conclusive than that which has hitherto led us to adopt the views which we have stated in the text.—Rev.

souffle is said to be more extended and louder; but any one who has had the slightest practice in obstetric auscultation will have observed that there are no two cases in which the souffle is exactly alike, even where there is but one child. In triplet pregnancy, the sounds must evidently be very remarkable. The powerful resonant souffle, the confused pulsations of *at least* two fœtal hearts to be heard at the same moment, must afford a most interesting subject of investigation to the auscultator. As a means of diagnosis in extra-uterine pregnancy, auscultation offers but little hope of being useful; and we confess that we should place much more faith in the general history of the case, and a careful consideration of the symptoms, aided duly by manual exploration, than in the stethoscope. Professor Hohl has entered upon the various signs of this extraordinary deviation in a very insufficient manner, and would, we think, have done much better if he had left them out altogether.

In ascertaining the position of the child, we may conclude with tolerable certainty that when we hear the fœtal pulse on the left side, the occiput will be turned towards the left foramen ovale (supposing that the child presents with the head), or, in other words, that the head will be in the first cranial position; and that where we hear it on the right side, we shall find the head presenting in the third, and passing into the second position. With these views we are glad to find that M. Hohl fully agrees; but he gives some others which are both new and curious, and, although of themselves highly interesting, still require further investigation. He considers that the placenta is almost always attached to that part of the uterus to which the anterior surface of the child is turned; so that, according to his opinion, we shall always hear the souffle in the opposite direction to the fœtal heart. With this view we cannot possibly agree; for, if it were correct, we should generally hear the souffle on one side, and rather backwards, instead of forwards, which is by no means the case; for we can hear it fully as often, nay more frequently, on the anterior wall of the uterus than elsewhere: it only tends to prove that the principle with which he set out, viz. that the souffle is only to be heard where the placenta is attached, is incorrect.

There can be no doubt but that the stethoscope offers a most invaluable means of diagnosis in cases where it is required to ascertain whether the child be alive or not. Few subjects are of greater importance, and yet few have been involved in greater difficulty and obscurity: even the sensation of a heavy weight rolling about the abdomen as the patient moves or turns, cannot be looked upon as a certain proof of the child's death. Several cases have occurred in Dr. Kennedy's practice, where, in spite of this symptom, on which so much confidence has been placed, the fœtal pulsation proved the child to be alive. We must confess that at one time we placed considerable reliance on this symptom, and, even although it had occurred two or three times where the result of the labour showed that the child was alive, still we looked upon these as rare exceptions to an otherwise undeviating rule. We believe that, to the *practised* auscultator, the stethoscope affords a *certain* means of ascertaining whether the child be dead or alive, inasmuch as when he is not able anywhere to detect the fœtal pulsations, he may rest satisfied with this negative sign, and conclude that it has ceased to exist: the souffle, becoming fainter, will also assist his diagnosis, but is not of such certainty, on account of the great variety of tones which it presents in different subjects. The usual list of symptoms which is given in works of midwifery is of little service: even taking them conjointly leads us to an uncertain diagnosis, and separately they are not worthy of consideration. The period at which it is generally most important to ascertain whether the child be alive or dead is *during* labour, when, from the swelling of the cranial integuments, we know with certainty that it must have been alive in the early part of labour, and where, from the difficulty of the case, we are unwilling to risk applying the forceps, without feeling assured that we are putting the mother to this danger and suffering for the sake of a living child. How often must it have been the lot of the accoucheur to feel the most painful uncertainty on this point, and to know that he must only be guided by the state of a mother's powers as to whether he should put her to the trial of a severe forceps delivery, when, if he could have determined that the child was dead, he might have lessened the head and quickly terminated her sufferings.

The diagnosis of the child's death by auscultation, as being a negative sign (the *absence* of the fœtal pulse), can only be available and trustworthy where the practitioner, by constant attention to the subject, has acquired such experience and tact as to make it nearly a matter of certainty that, if there be a fœtal pulse in the uterus, it cannot escape his ear. There are probably cases where, from a variety of circumstances, it will be rendered difficult, and at times uncertain; but day after day convinces us that these difficulties and sources of uncertainty will in great measure disappear, in proportion as our experience and correctness in ausculting increase. The observations of Dr. Kennedy on this subject are of great importance and merit, and afford a striking example of what the stethoscope can do in experienced hands.

Where almost every acknowledged sign of the child's death has been present,—when, in all her previous labours, her children have been born dead, and, from such a repetition of all her former symptoms, the patient has felt convinced that she should be again exposed to the disappointment of bearing a dead child,—the stethoscope has brought to her the glad and certain tidings of her infant's life, and the result of her labour has proved the correctness of its diagnosis. Even when the prolapsed cord has been felt without pulsation, or only with such an uncertain trace that it could be scarcely felt by the finger, the fœtal pulse has been heard, the forceps have been applied, the child delivered, and resuscitated. On the other hand, where the general appearance of health, the firm full breasts, the entire absence of uterine flaccidity and sensation of a weight rolling about the abdomen, and every other symptom indicated that the child was alive, the absence of fœtal pulsation has warned the accoucheur of its death, and the labour has been terminated by the birth of a dead child, which, from its appearance, had ceased to exist for some time previously. These are facts which we earnestly recommend to the consideration of our professional brethren.

Professor Hohl has given some observations on morbid states of the ovum as indicated by the stethoscope; viz. that in cases where, during labour, the souffle has been remarkable for its resonant piping sound, he has found the placenta full of little deposits of phosphate of lime; and that, in other cases, where the patient has complained of a burning pain at one side of the uterus, he has heard the souffle of a peculiar tone, and has found the placenta hypertrophied or otherwise changed. These, and other observations of a similar nature, must be for the present received with due caution, and we refrain therefore from giving any opinion upon them. His observations on inflammation of the amnion are, we think, rather hazardous. The remarks on the use of the stethoscope in asphyxia of new-born children contain nothing new. An interesting case or two of the sort are given in illustration; but we infinitely prefer the observations on this subject by Dr. Kennedy, in his work published almost immediately after the volume we have now been reviewing.

Professor Hohl's second volume is divided into two parts,—*das Sehen* and *das Fühlen, (seeing* and *feeling,)* or, what we may Latinize by *Exploratio per Visum, et per Tactum.*

We need scarcely observe, that both of these are subjects of great importance to all branches of the profession, but essentially so to those practising midwifery. The experienced eye is not less necessary—we may say invaluable—to the practitioner, than a similar correctness of the ear and of touch, and frequently, at the first glance, suffices to help him on some considerable way in forming his diagnosis. M. Hohl has most appropriately quoted from a French author, "Le visage est un livre ouvert, ou les autres peuvent lire a chaque instant ce qui se passe dans notre âme." This observation is not confined merely to the face: with a medical man, it extends to the whole figure of the patient,—her gait, carriage, manner; all of which require a short but scrutinizing glance, previous to our entering on a more minute investigation. "The surrounding objects," as Professor Hohl remarks, "ought not to pass unobserved: they exhibit the circumstances under which the patient is living, her occupations, mode of life, &c."—"It is of the greatest importance to pay attention to the peculiar constitution and habit, the age and temperature of the individual: it is a field, as Naegelé observes, still capable of much cultivation; and, the more attention it receives, the more important and valuable will be the fruits."—"The experienced eye will not find much difficulty in forming a tolerable estimate of the patient's age, and in ascertaining her habit and general temperament: it must scrutinize the whole form and character of her person; its proportion, size, precocious or tardy development." We should go on with the description of the general external appearances of a patient where the pelvis is deformed, but we prefer giving our readers the original passage from Professor Naegelé's "Lehrbuch," from which it was evidently taken.

"The chief symptoms are, the lower jaw projecting beyond the upper, and a very prominent chin; teeth transversely grooved; unhealthy appearance; pale ashy look; diminutive stature; waddling gait; the chest thrown back; the arms hanging behind; the abdomen projecting; distortion of the spine and breast; thick wrist and ankle joints. Curvature of the extremities, especially the lower ones, where the spine is straight, is of great importance: where the lower extremities are crooked, the pelvis is generally faulty." (§ 144.)

"When we see," observes M. Hohl, a little further on, "the light but firm step of the female figure, we naturally conclude that the inclination and size of the pelvis are in the proper degree; that the uterus and parts of generation have the normal position, &c." We know the look of a deformed person; but her peculiar cast of features, the anxious and generally astute expression of her countenance, is a character of face not so frequently observed where the pelvis is deformed as where there is curvature of the spine; and we need scarcely add, that the one species of deformity is *not* frequently accompanied by the other. The general character and appearance of a woman with a narrow pelvis has been already sufficiently marked in the short but graphic description of the distinguished Naegelé, and we cannot add to it without diminishing its merits.

The practised medical man will instantly detect, as he enters the room, the flushed face and anxious eye of the patient, the suspicious-looking dram bottle in the corner, the heavy load of bedclothes; or, in other cases, the pale face, the sunken eye, the expression of exhaustion, the eager longing for cooling drinks, the fruitless pains. Even if she be asleep, the first glance will detect the half-closed eye, the restless muscles of the face, the uneasy posture: he will instantly perceive if her colour be of its natural degree, the features swollen or collapsed. M. Hohl thinks that we may even distinguish *primiparæ* at sight; but this would be rather a hazardous species of diagnosis in this country, especially among the upper ranks, where the youthful freshness of form and colour are so remarkably preserved. We perfectly agree with him, nevertheless, that no point of observation should be overlooked or thought lightly of: the most (apparently) indifferent objects are capable of becoming valuable, when we least expect them, and only serve to prove that we cannot watch nature too closely. Although we may not be able to distinguish a primipara by her look, yet there are other points connected with labour, where an attentive glance will tell us a great deal. How often, on entering the room during a pain, have we not instantly felt assured, from the manner and expression of the patient, that the membranes had burst, that the head had passed through the os uteri, and entered the vagina? The tone of the voice, it is true, assists in these cases, and ought not to be overlooked. An experienced observation is of infinite use in cases where any attempt is made at imposture, or where the patient attempts to conceal the commencement of her labour: the slight involuntary start, the half-suppressed shrinking, the change of countenance, &c. can seldom escape an attentive observer. In prisons, and other public institutions, its absolute necessity is felt daily.

At pages 20 and 21, we meet with observations respecting obliquity of the uterus, as a cause of malposition of the child, which, in a work like the present, that, despite of its prolixity, we must in justice say contains much valuable information, we had not expected. It is not fair to the character of the German obstetricians to allow this to pass over without notice, because we can, with equal pleasure and satisfaction, assure our readers that they, for the most part, entirely oppose this old, incorrect, and long since disproved theory; a theory which was directly contradicted by Chapman, now exactly a century ago, and has since been distinctly shown, not only by Ould, but also by Boer, Mursinna, Dewees, &c., to exist merely in books, and not in nature. We are sorry that Professor Hohl should have retained such a worthless remnant of by-gone errors, more especially as he is evidently well acquainted with the mechanism of parturition, and appears to be a man of considerable experience.

We pass over some unimportant remarks respecting the discoloration of the linea alba, as a sign of pregnancy, in which little or no confidence can be placed; and will give his own words, where he speaks of the areola.

"The immediate vicinity of the nipple, viz. the areola, is very variously coloured. Where the skin is delicate and white, I have seen it of a rosy red colour, like the lips; and this is more frequently the case with *primiparæ* than with those who have already borne children; but we also occasionally see it brown, dark brown, sometimes even almost black. I have seen it at times covered with small hairs: only one patient remarked that she had never observed this appearance before; but, whether these hairs fall off afterwards or not, I cannot say. The areola varies also in size. I have seen it forming a ring scarcely half an inch in breadth round the nipple. Generally speaking, however, it is broader, and I have met with cases where it was even two inches in breadth. The margin is mostly well defined, but occasionally it passes irregularly into the white colour of the surrounding skin. I have seen women, who had never had children, where the colour of the areola underwent a change at each catamenial period."

There has been a good deal of discrepant opinion respecting the correctness of the areola as a sign of pregnancy, chiefly, we suspect, owing to a little want of judgment on both sides. We believe that, in a young and tolerably fair woman, pregnant for the first time, the change in the colour of the skin round the nipple is very distinct, and that this may be a sign on which we may rely with considerable confidence; but, in dark swarthy complexions, and especially in those who have borne already, it seldom regains its former colour, except perhaps in the genuine blondine. With regard to the nipple being as it were inflated, as Rœderer describes it, this may be all very true: we have seen it occasionally; but in this country, at least, the presence or absence of this sign must not be a guide: the pressure of the dress is too serious an obstacle to its regular development to allow the display of its true character.

At page 28, M. Hohl enters into a minute detail of the hypotheses which the ancients entertained respecting the means of prognosticating as to which sex the child was to be of. We cannot help expressing our surprise, as well as regret, that, in the nineteenth century, an author should seriously discuss the various (and to many we may add, disgusting,) theories which have been broached in former times of ignorance and superstition, not only respecting the signs by which we may foretel the sex of the child, but also the means to be taken for securing its being of the one or other sex at pleasure. Our readers will scarcely believe that all the old notions of Hippocrates, Moschion, Albertus Magnus, &c., respecting the peculiar powers of the right and left testis and ovary are gravely discussed, and works quoted, the very titles of which ought to be a sufficient veto to their being introduced into the pages of a book like this. The influences of the moon, before or after the full, are seriously considered; and even a couple of pages are devoted to a long series of observations on this point by a Dr. Löwenhard, with the prudent conclusion on the part of the author, that the moon has no effect in determining whether the children are to be boys or girls. Professor Hohl should have remembered the good old adage, "*aus Nichts kommt Nichts;*"—ex nihilo nihil fit;—for he would have added not a little to the value of his book by leaving out the whole subject. Respecting the effects also of the moon on conception, we must give a similar opinion.

We would not be supposed to be indifferent to these interesting but mysterious points of physiology: we merely complain of the manner in which the subject is treated, and the quantity of worse than useless matter with which he has thought necessary to load his book. The only rational attempt to solve the question is by the celebrated Carus, of Dresden, in his "Gynakologie;" and even he has touched upon it more in the way of hint than serious discussion. Carus is disposed to think that a predominance of vital energy in one of the parties, at the moment of conception, has an influence in deciding the sex of the embryo; and this has been the prevailing opinion among men of rank in their profession in this country. It is fair, however, that we should state M. Hohl's own opinion on this point,—more especially as it is given with no little confidence, and several cases are mentioned, the result of which certainly agrees with the data upon which he founds his prognosis.

"A woman is mostly pregnant with a male fœtus when her colour remains quite natural; except, perhaps, where it is somewhat changed by small brown spots upon the forehead and about the mouth; when the linea alba either divides the abdomen by a narrow brownish yellow line into two halves, or where this appearance is entirely wanting, and the umbilicus retains its usual colour. On the other hand, she is pregnant with a female fœtus, where the colour of the face is either generally altered; where she has the look of a patient suffering under disease of the liver; where large spots are observable upon the forehead, like freckles, extending across the bridge of the nose, and forming a circle round the mouth, or merely bound the edge of the upper lip; or when these spots appear distinctly only at one or only some of the above-mentioned points. The linea alba is here of a bright brown or yellowish colour, and appears much broader than in the other case: the navel, and its immediate vicinity, are also generally more coloured than where she is pregnant with a male fœtus. Freckles and other spots upon the skin, as also the irides, retain their colour during pregnancy, where there is a boy; but change colour, and become more distinct, when the fœtus in utero is a girl: this," he adds, "is a certain rule."

At page 57 there is a species of note, or what we should have rather called *digression*, had it not been prefaced by three questions relative to the above quotation, which he professes to answer. The questions are as follow:

"1. How do we explain the discoloration which takes place in pregnancy?

"2. What is the connexion which exists between the varieties of this discoloration and the difference of sex?

"3. If this connexion can be proved to exist, can the production of the one sex or the other be influenced?"

The explanation, which runs on for forty pages, and that too in a smaller type than the rest of the letter-press, is a strange medley of hypothetical reasoning, strained beyond all bounds to meet the wished-for point: viz. how to prognosticate the sex of the child. In the course of these observations, he gives the reader some interesting tables of 195 cases, in which the age at which the catamenia first appeared, and the manner in which this took place, are noted. Tables of this sort, if drawn up with accuracy, which these appear to be, are always valuable; and with this view we venture to inser- them; but the utility in the present instance is considerably dimit nished by our being ignorant of the peculiar habits, constitution, &c. of the individuals from whom the observations were furnished.

1.

The Catamenia first appeared

| | | | |
|---|---|---|---|
| 1 | at the age of 12 | in 3, | with derangement of health in . . 0 |
| 2 ... | 13 ... | 10 | ... 3 |
| 3 ... | 14 ... | 27 | ... 3 |
| 4 ... | 15 ... | 33 | ... 10 |
| 5 .. | 16 ... | 41 | ... 5 |
| 6 ... | 17 ... | 28 | ... 5 |
| 7 ... | 18 ... | 21 | ... 4 |
| 8 .. | 19 . . | 11 | ... 4 |
| 9 ... | 20 ... | 12 | ... 7 |
| 10 ... | 21 ... | 5 | ... 4 |
| 11 ... | 22 ... | 2 | ... 1 |
| 12 ... | 23 ... | 1 | ... 1 |
| 13 .. | 24 ... | 1 | ... 1 |
| | Total | 195 | |

4.

The Catamenia returned

| | | |
|---|---|---|
| 1 | every 14 days ... | in 5 cases. |
| 2 ... | 3 weeks ... ... | 43 |
| 3 ... | 4 ... ... ... | 138 |
| 4 ... | 6 ... ... ... | 1 |
| 5 ... | 2 to 4 weeks ... | 2 |
| 6 ... | 3 . 4 ... ... | 2 |
| 7 ... | 4 . 12 ... ... | 1 |
| 8 ... | 4 . 18 ... ... | 1 |
| 9 ... | 6 . 10 ... ... | 1 |
| 10 ... | 8 . 12 ... ... | 1 |
| | Total | 195 |

2.

The Catamenia were

| | |
|---|---|
| Profuse .. | in 83 |
| Moderate .. | . 71 |
| Sparing .. | . 40 |
| Variable ... | . 1 |
| Total | 195 |

3.

The Menses continued

| | | |
|---|---|---|
| 1 ... | 1 day ... | in 2 cases. |
| 2 ... | 2 days ... | . 21 |
| 3 ... | 3 .. ... | . 54 |
| 4 ... | 4 .. ... | . 18 |
| 5 ... | 5 .. ... | . 11 |
| 6 ... | 6 ... ... | . 6 |
| 7 ... | 7 ... ... | . 2 |
| 8 ... | 8 ... ... | . 39 |
| 9 ... | 9 ... ... | . 1 |
| 10 ... | from 1 to 2 days in | 1 |
| 11 ... | ... 2 . 3 ... | 7 |
| 12 ... | ... 2 . 6 ... | 1 |
| 13 ... | ... 3 . 4 ... | 10 |
| 14 ... | ... 3 . 5 ... | 2 |
| 15 ... | ... 3 . 6 ... | 1 |
| 16 ... | ... 3 . 8 ... | 1 |
| 17 ... | ... 4 . 5 ... | 4 |
| 18 ... | ... 4 . 8 .. | 1 |
| 19 ... | ... 5 . 6 ... | 2 |
| 20 ... | .. 5 . 7 ... | 1 |
| 21 ... | ... 5 . 8 ... | 4 |
| 22 ... | ... 6 . 8 ... | 5 |
| 23 ... | ... 8 . 10 ... | 1 |
| | Total | 195 |

The rest of these forty pages of small print is quite beneath notice; and we must only request the English readers of this work not to judge of the state of obstetric science in Germany by what they find in this portion of M. Hohl's work: they must not suppose, for instance, that it is a prevalent notion in Germany that women contain more hydrogen and venous blood than men, and that male children are begotten in consequence of the female parent being in a state of positive electricity. It is always praiseworthy to investigate the hidden mysteries of nature, as far as possible; but this is not the way to set about it. The fact, that in hot climates the number of female children born preponderates, and in cold climates the contrary, is curious: but we must not lose ourselves in reveries, and fancy that we are thus investigating truth.

The chapter on Diagnosis per Visum is concluded with a few observations concerning the child. The appearance of the funis, and the changes it undergoes from the moment of birth until it is thrown off, are minutely described; and, as we do not recollect having seen them so well given elsewhere, we add a short translation of the passage.

"The new-born child presents several evidences of its recent birth, and the period at which this took place: we will, however, confine ourselves merely to the appearance which the funis, the skin, the eyes, and meconium present. When it ceases to be a fœtus, on account of ligature and division of the funis, the pulsation will continue to be felt in the fœtal extremity of the cord, for a space of from eight to ten minutes, according as the breathing has been fully established or not. Where this portion of the funis is in contact with the skin, the latter surrounds it like a ring, having the same colour with the skin in other parts.

"This ring at first is reddish, after a little is somewhat white, but soon becomes of an inflammatory red. This redness remains from twelve to twenty-four hours after labour; disappears and appears again in the course of sixteen or thirty-six hours. The ring gradually retracts, becomes deeper at its lower half, and forms at the upper part a thick crescentic edge. The funis now appears to spring from a hollow, in which a ring of pus, with a red margin, surrounds it at its insertion. This portion of the cord is at first white, here and there of a bluish tinge, and still firm. In the course of twenty-four hours after birth, (seldom before, and never after,) it begins to be flaccid. By the sixth day it has become dry; seldom sooner. It is now flat, transparent in places where there are no vessels; for, where these pass, we can distinguish streaky lines, which are not transparent. At last it falls off; the surface, to which it was attached, is covered with pus, along the edge of which a number of little bloody, or only bright red, points may be observed; the spot to which the cord was attached looks like a large pea at the bottom of the hollow, the surface being drawn downwards and inwards, so that the upper part of the circle passes into the skin; whereas the lower portion forms a small crescentic fold, which partly conceals the hollow."

The description of the appearance of a premature child, as far as it goes, is very fair; but Professor Hohl has left out two or three important points of diagnosis which ought to have been noticed: for instance, in male children, which have been carried the full time, the scrotum is corrugated, not peculiarly red, and generally containing the testes; in female children, the nymphæ are covered by the labia: whereas, as Professor Naegelé observes, in premature children the testes are not always down, the labia are apart, and the nymphæ protrude; and in both sexes the external generative organs are remarkably red.

As to the appearances which a child puts on when born *too late*, this is rather beyond our comprehension. The whole is described as regularly as if the partus serotinus were a thing of every-day appearance, instead of being a fact the correctness of which has been questioned by the highest authority. The quotations which our author has given in a note from the Gardner Peerage cause might have been spared: they have been long since viewed in their proper light in this country.

The second, and by far the largest portion of this volume, is devoted to *Das Fühlen*, or what, as already said, we may call "*exploratio per tactum*"—(*le Toucher* of the French.) The importance of this subject cannot be impressed too forcibly on the mind of a young practitioner: it is not sufficient that he should be merely able to feel the os uteri, and ascertain its degree of dilatation during labour, or to satisfy himself that the head presents because he feels a large hard mass beyond:

"He must learn to know," observes Professor Hohl, (p. 117,) "the deviations which result from variety in the size, constitution, fibre, and age of the person, the posture of the body, and particularly the inclination of the pelvis. He should minutely investigate the changes produced by pregnancy and labour, and make himself acquainted with the natural condition of the parts which form the subject of obstetric examination, as respects their hardness or softness; their elasticity, form, and shape; their position, direction, roughness or smoothness, moisture or dryness, and their temperature. Let him learn to distinguish the changes which these parts present before, during, and after menstruation; during pregnancy; during and after labour; their characters in health and disease; the appearances they present after the change of life in women who have had children, and in those who have not. Let him learn to ascertain the weight, and mobility, and bulk of the uterus; the various movements of the fœtus; the different parts of it, their position, and the parts belonging to the fœtus."

His enumeration of the various questions and points of diagnosis which the accoucheur will be called upon to solve by manual exploration, is perhaps necessarily long; the nature of the subject demands it, and, if our limits would have permitted, we would have willingly translated it for our reader's perusal. We pass by a quantity of what the author calls general rules; such as, how the practitioner is to behave, what preparatory arrangements he is to make; that he is not to pull off his coat, or to stare at his patient in the face whilst examining, except it be under suspicious circumstances; that he should carry a box of pomade in his pocket, &c.; the greater part of which we shall beg to leave under the head of what a highly respected friend of ours upon the continent would term "*Kram.*" It is useless to spin out long rules for the behaviour and conduct of a practitioner in these cases: he must behave with honour and with delicacy, and, if he cannot do so, the sooner he quits his profession the better. Many of the author's directions for performing the external and internal examination are quite impracticable, and we are confident would be as little tolerated by the women in Germany as in England. With regard to the position in which the patient is to be examined, this, of course, will vary according to circumstances which are self-evident. In cases of organic disease, displacement, and in the early months of pregnancy, it is desirable that she should be examined standing. During or shortly after labour, and in cases of premature expulsion, &c., it will be necessary that she should be in the horizontal posture, and generally this is most convenient when she is upon her left side. The only rule which we have found it necessary to give a beginner, when examining a patient standing, is to hold the forearm as perpendicularly as possible, so that the elbow is completely covered by the clothes as he kneels before her, and directly under the os externum. The hand should be strongly supinated: by this means the index or examining finger passes along the posterior edge of the vulva, whilst the other fingers and thumb occupy the pubal arch, and thus allow the operator to reach much further than he otherwise would do.

With regard to measurement of the pelvis by instruments, whether externally or internally, we shall not stop to consider the various pelvimeters which have been invented at different times; they have, as a celebrated teacher of midwifery truly says, only one fault, viz. that of being perfectly useless. Baudelocque's callipers, (*Compas d'Epaisseur,*) deserves perhaps a better character: it is certainly a useful instrument to determine the external distance from the sacrum to the pubes. Thus, in a well-made woman, it should measure seven inches: we subtract two inches and a half for the thickness of the sacrum and the soft parts covering it, and half an inch for that of the os pubis; thus leaving a remainder of four inches, which is the antero-posterior diameter of the pelvic brim. But, besides a variety in the thickness of the hard and soft parts, whether natural or from disease, it is exceedingly difficult to determine correctly the exact point where the sacrum begins, and a slight obliquity of the instrument either above or below this point will give a very considerable error. The best pelvimeter is the finger of an experienced accoucheur: by constant practice, he knows exactly what portions of the pelvis he can reach easily, with difficulty, or not at all: he familiarises himself with the precise shape of the bones which form its parietes, the curve which they present, the angle at which they stand, and their relative situation to each other: he accustoms himself to measure by the touch the thickness of the soft parts which the pelvis contains, their rigidity, firmness, or relaxation, the degree of swelling which they may have undergone during labour, &c.; and thus enables himself to estimate not only in what degree the pelvis itself is at fault, and what part of it, but also in how far the diminution of space is to be attributed to the condition of the soft parts. The manner in which the head enters the superior aperture will frequently of itself show that the pelvis is contracted, and afford a tolerable notion of the degree in cases of considerable deformity; it is then very easy to measure the precise distance from the promontory to the pubes upon the examining finger.

In speaking of the subjects for the investigation of which the external and internal manual exploration are employed, M. Hohl has arranged them under eight heads: viz. 1st, the examination to determine cases of doubtful sex; 2d, to decide as to the existence of virginity; 3d, the examination in cases of rape; 4th, to determine the possibility of sexual intercourse and conception; 5th, the existence of pregnancy; 6th, examination to investigate various points during labour; 7th, examination of a pregnant female after death; and 8th, the examination after labour.

With regard to the second and third points, viz. ascertaining the existence of virginity, and proving the commission of rape, we can prove little by mere manual exploration. The presence or absence of the hymen has long been ascertained to be no evidence whatever for or against the existence of virginity: a case occurred to us, about a year ago, of a primipara whom we attended during labour, where the greater part of the hymen was still existing. The different conditions of the labia, nymphæ, and vaginal rugæ, are much too uncertain to ground any decisive opinion upon. A slight degree of dyspepsia or other abdominal derangement, or of leucorrhœa, will produce nearly all the changes which M. Hohl considers necessary to observe; and, as to feeling whether the clitoris be still covered by its preputium, we need not make any observation upon it. Few decided changes take place in the female external organs from only one occurrence of sexual intercourse, and little appreciable alteration is produced until the passages have undergone the extreme dilatation which they suffer during labour. As to the changes to be observed by manual examination in cases of rape, our author is right enough when he says that, unless this be performed soon after the commission of the crime, our examination will lead to little or no result; and the difficulty will be much increased if sexual intercourse had previously taken place.

The fifth head, viz. the diagnosis of pregnancy, is, or ought to be, much more interesting, and is a subject which of all others should be handled in a clear, concise, and practical manner; but unfortunately we have to wade through the same excessive verbosity. He divides it into nine questions, on the importance of which we fully agree with him; they are as follows: 1st, is she pregnant? 2d, in what month? 3d, is she pregnant for the first time? 4th, is there more than one child? 5th, is a state of disease combined with pregnancy? 6th, is it extra-uterine pregnancy, with or without uterine pregnancy? 7th, is the fœtus alive? 8th, what is its position? 9th, will the labour be anormal from mechanical obstruction?

In determining the first question, we will not detain our readers with the long and tedious enumeration of the various points to which the practitioner must direct his attention, and upon which the changes are rung with a degree of persevering repetition that is almost exhausting. It is little short of nonsense to suppose that the state of the os externum and the carunculæ myrtiformes, the calibre of the vagina, the swelling of its parietes, its temperature, secretion, the length and condition of its rugæ, are points on which we can fix the slightest data for forming our opinion as to the presence of pregnancy. We cannot agree with him that the temperature or secretion of these parts are so much increased during pregnancy: with respect to the former, we might say the contrary; for we have repeatedly found the vagina of a pregnant woman impart a feeling of coolness to the finger, and it must be a well-known fact to every body who is frequently in the habit of examining per vaginam, that the labia are frequently even cold: this is in some measure produced by the moisture of the vaginal secretion, but we cannot think that this is so increased in quantity during the earlier months of pregnancy as in any degree to justify its guiding our prognosis. This certainly applies to the relaxed females of a great metropolis, or those living in the swampy parts of Holland, &c., where it is a known fact that there is much greater disposition to copious vaginal secretion than elsewhere, but we apprehend that the insufficiency of this as a point of diagnosis applies equally to the more robust natives of other districts. We have examined many hundreds of the author's countrywomen during the last months of pregnancy, as well as at other periods, and are not inclined to make an exception in their favour.

In examining a woman to ascertain the existence of pregnancy, it is desirable to place her in such a posture, that we may examine both externally and internally at the same moment, and also ensure, as far as possible, the complete relaxation of the abdominal integuments. Some excellent directions have been left us for this purpose by Rœderer, which have been also quoted by the late W. J. Schmitt, of Vienna, in his short but valuable collection of doubtful pregnancy cases. "After the third month, the uterus projects above the pelvis, gradually increases and distends the abdomen; but a careful examination is necessary, in order to distinguish the enlarged uterus from other prominences, because an enlargement of the abdomen from disease may easily be confounded with pregnancy: merely looking at the abdomen will not assist us much in our diagnosis; we must examine by the touch. In order to prevent any chance of uncertainty, the following points should be attended to: we should place the patient upon her back (before she has taken her meals, and having previously emptied the bladder and rectum,) with the head and feet raised above the loins, the heels drawn up to the nates, so as to relax the abdominal parietes; the practitioner should place his hand across the abdomen, so that the little finger is turned towards the pubes, and thumb to the navel. Let the patient breathe deeply, and the practitioner press gently with his hand during expiration; if he feels at this moment a hard globular resisting mass above the pubes, he may be certain that this is the enlarged uterus."

In ascertaining how far pregnancy is advanced, our attention must be chiefly directed to the circular form of the os uteri, its being closed, the smoothness and softness of its edges, (now no longer lips,) the alteration in the shape, size, and substance of the *portio vaginalis;* viz. that part of the cervix which projects into the vagina; a distinction which is very useful, and which we have for some years adopted from the German accoucheurs; the increased size, weight, and diminished mobility of the lower portion of the uterus; and lastly, if it be in the latter months, the contents of the uterine cavity and diagnosis of the presenting part: this must also be combined with the external examination of the abdomen, in order to estimate the height of the fundus above the symphisis pubis, the size and form of the uterus generally, and whether the movements are yet perceptible: these are the chief practical points of investigation to which the practitioner must turn his attention in such cases: but, as to the old, oft-repeated dogma of its being necessary to examine the puffiness or turgescence of the vaginal parietes, the diminution in the size and number of its rugæ, the prolapsus-like duplicatures of the anterior wall, its temperature, mucous secretion, &c. &c.,—all this is, at least, useless in practice.

In deciding whether it be her first pregnancy, our chief attention must be directed to the form and condition of the os uteri. An os uteri which has once undergone the dilatation which takes place during labour, seldom entirely recovers its former shape: it becomes unequal, so that, instead of forming a circular depression, with edges quite smooth, like a dimple, as it were, at the end of the cervix, it forms an irregular-shaped margin, with uneven edges, which are generally hard in places, from the little cicatrices of former labours. These are important points of diagnosis, and have more than once enabled us to assert confidently that the patient had already borne a child, in spite of previous assurances to the contrary. The absence, however, of these effects of parturition,—we mean the perfectly round and smooth depression of the os uteri, as felt in the primipara,—is not always a proof of first pregnancy: we have occasionally, though rarely, met with a similar condition in a patient who had already borne a child. Besides the examination of the os uteri, that of the perineum, and especially its frœnulum, should not be neglected; for this latter rarely escapes being somewhat torn in the first labour. As regards the external examination in determining whether it be her first pregnancy or not, the flaccid abdomen and rugæ in the skin are certainly effects of previous labour which are worth noticing; but it must be recollected, that their presence or absence are not distinct proofs for or against.

Amid the poverty of materials which pervades many parts of this prolix work, we had looked with no small degree of hope to the diagnosis of pregnancy, when connected with a state of disease. The reader will judge of our disappointment on finding the whole of this highly interesting subject comprehended in a space of barely one page. The subject of extra-uterine pregnancy receives a similar allowance of space; but the interesting question as to whether the child be alive or dead before labour is disposed of in ten lines. It appears to us that the author has made a grand mistake, in discoursing, page after page, as to *how* we are to examine under different circumstances, and *how* we are, in this or that case, to attend to the size, and dilatation, and temperature, &c. of the various parts; but the *what* we are to find, and the precise nature of the changes produced, he has somehow managed to leave almost entirely unnoticed.

Under the question as to what is the position of the child, he informs us (as of something original,) that, in face cases, the face, in all probability, does not present until the head begins to enter the superior aperture: this has been long suspected, and we recollect having our attention called to this subject, at least seven years ago, by Dr. Breitenbach, of Heidelberg. When the head presents at the brim of the pelvis in the first position,—viz. the occiput forwards and to the left,—if by any cause the occiput should be prevented descending into the cavity of the pelvis, or the forehead experience unusual facility in entering it, the head will turn upon its transverse diameter, and the forehead, followed by the face, will sweep past the right sacro-iliac synchondrosis, and occupy the pelvic cavity, while the occiput still remains at the brim: in other words, the first position of the head becomes the first position of the face; and the frequency of that face presentation where the chin is turned towards the right side of the pelvis, (first position of the face,) in proportion to those where it corresponds to the left side, (second position of the face,) tends not a little to confirm this view. All originality on this point we must decline conceding to M. Hohl. His rules for ascertaining a case where the nates present, are insufficient and incorrect: it is true that the nates do not descend so far into the pelvis before labour as the head usually does, especially in primiparæ; but this is no proof of a breech presentation: the shoulder may present, and in this case we shall certainly not be able to feel the presenting part until labour has commenced; or the pelvis may even be deformed, and the head presenting, but unable to descend through the brim.

The investigation of various points of interest during labour he divides under the following questions: 1st. Whether labour has really commenced, and will go on? 2d. How far it has advanced? 3d. What are the obstructions to labour? 4th. Is there a second child in the uterus? and 5th. Is the child alive or dead? In the consideration of the second question, viz. how far labour has advanced? our author suddenly rouses himself, and offers some really good observations respecting the diagnosis of this point in premature expulsion.

"Attention to this question," says he "is of especial importance, where abortion threatens to take place; because our practice will be considerably influenced by it; our hopes or despair of averting the expulsion will depend upon it. When the appearance of hæmorrhage, with periodical hardness of the uterus, relaxed mammæ, and fallen abdomen, afford reason to dread expulsion of the embryo, the internal examination must be instituted with the greatest caution and gentleness: it will be chiefly directed to the vaginal entrance, the vagina, and the uterus; especially the os uteri. With respect to the former, this, as abortion proceeds, will be felt somewhat wider, from participating in the cushiony and soft condition of the vagina, in which we shall find an increase of mucous secretion, (in all probability, more or less mixed with blood,) the temperature increased, and coagula lodging in it. Not unfrequently the anterior wall of the vagina will be felt peculiarly swollen; and, if there be any difficulty in passing water, we shall feel a long bolster-like mass, which is the swollen urethra. The uterus sinks somewhat lower in the vagina; we feel the external os uteri (os tincæ); and sometimes the os uteri internum also open. Where the opening is large enough to admit of the tip of the finger, it feels as if surrounded by an elastic ring of cartilage; where this is the case, the os uteri seldom closes again: in other cases it is more dilated, and we can feel the ovum presenting. When the abortion is in the second or third month, the practitioner must bear in mind that it may have been retention of the menses, and that therefore what he feels in the os uteri may either be an ovum or a coagulum of blood. To decide this point, he must keep his finger in contact with the substance lying in the os uteri, and wait for the accession of a pain, (for where clots come away, pains like those of labour are present,) and ascertain whether the presenting mass becomes tense, advances lower, and increases somewhat in size; this will be the case where it is the ovum pressing through the os uteri. On the other hand, if it be a coagulum of blood, which it is well known assumes a fibrous structure, it will neither become tense nor descend lower, but be rather compressed. Generally speaking, the ovum feels like a soft bladder, and at its lower end is rather round than pointed; whereas a plug of coagulum feels harder, more solid, and less compressible, and is more or less pointed at its lower end, becoming broader higher up, so that we generally find that the coagulum has taken a complete cast of the uterine cavity. If we try to move the uterus by pressing against this part, it will instantly yield to the pressure of the finger if it be the ovum; whereas the extremity of a coagulum, under these circumstances, is so firmly fixed, that, when pressed against by the finger, the uterus will move also. When abortion happens at a later period of pregnancy we shall be able to feel the different parts of the child as the os uteri gradually dilates, viz. the feet, or perhaps the sharp edges of bones, although we cannot distinguish the form of the head, from the cranial bones being so compressed and strongly overlapping each other."

We pass over some observations on mole pregnancy, and on the diagnosis of the various causes of obstruction which may occur during labour: both are ample fields for interesting information, but, unluckily, either our author does not always possess it, or, at least, has not the power of communicating it. His observations upon the signs of the child's death *during* labour are excessively meager and incomplete: nor does he even enumerate half of those symptoms which are allowed on all hands to exist. The diagnosis of the child's death before, and especially *during* labour, has long attracted our attention, from its great difficulty as well as importance. The wrinkles of the scalp, gradually passing into the well-known *caput succedaneum,* or cranial swelling, of course show that the child is alive: we know also that the flabby scalp, without any swelling whatever, and sliding, like so much wet leather, over the skull; the edges of the bones remarkably sharp, everted, and grating against each other; and, in some cases, the crackling feel of emphysema under the skin, from putrefaction, are indications of the child's death. But, take the most frequent case where it is important to ascertain if the child be alive or not,—viz. a difficult labour, where the head is large, or the pelvis somewhat contracted, and, when called, we find the soft parts more or less inflamed and swollen; a case where the forceps may be advisable, and perfectly practicable, but where, from the state of the patient, it would be highly desirable to release her at once by perforation, could we be sure the child is dead;—what signs have we to guide us in this respect? The child was evidently alive at the beginning of labour, by the large œdematous tumour upon the presenting part of the head: we can scarcely reach a suture on this account, and yet, from the long duration and severity of the labour, it may have ceased to exist some hours:—how are we to prove this? It is here that the stethoscope shows its real value: if the child be alive, we shall most surely hear its circulation;—but is the absence of this sound a proof of the child's death? We believe (as we before observed,) that, in the hands of a practised auscultator, the negative sign of *not* being able to hear the fœtal heart after a careful examination, may be taken with tolerable certainty. This is a subject which we beg to recommend most earnestly to the notice of our professional brethren: its importance, both as regards the mother and her child, needs no comment, and must be our apology for the repetition. We are glad to see that Professor Hohl agrees entirely with the views of the late W. J. Schmitt, of Vienna, on the subject of hour-glass contraction, (p. 235.) "Of the many cases of incarceratio placentæ which have occurred to me, I have always met but one opening, viz. the natural os uteri, and have never seen a second opening formed by stricture of the body of the uterus, and leading to a second cavity; and I have perfectly convinced myself that, from the height of the uterus at this time in the pelvis, and from the dilated state of the upper part of the vagina, we may be very easily induced to suspect stricture of the uterus."

In describing the changes which take place in the uterus and vagina after labour, we will again quote M. Hohl's own words, (p. 241:)

"At the moment of the placenta being expelled, we feel from without the fundus gradually diminish and sink lower; it still projects beyond the pelvis; we even feel the whole uterus like a ball, still high, and somewhat to the right side. Where this is the case, we generally find the vagina somewhat elongated. If we do not examine the abdomen till a few days after labour, we find the uterus more diminished; and, in the course of some weeks, it has returned to its former dimensions. Immediately after labour, the vagina is soft, swollen, and covered with mucus, the temperature increased, the canal itself dilated. After some four or six weeks, it becomes narrower, the rugæ reappear; but they are further apart, and the edges of the separate folds are not so well defined as in the virgin state. The greater the number of labours she has had, the smoother the vagina becomes, and the longer is the time before it contracts again. It is only now and then that we feel here and there a ruga; the portio vaginalis is flaccid; the edge of the os uteri lobular, hanging down into the vagina and still open. In the course of a few weeks, we first begin to feel the portio vaginalis recovering its original shape, and the mouth of the uterus closed; the swelling of the urethra, which is usually felt immediately after birth, gradually disappears."

Does the uterus, *immediately* after birth, gradually contract, and its fundus sink behind the pubes, until it has nearly returned to its former size? We feel bound to meet this with a direct negation. From the results of our own observations, and from those of one, in whose experience and accuracy we place the greatest confidence, the changes in the size of the uterus after birth are as follows:—After the state of firm contraction in which we feel it, immediately after the completion of labour, when it is felt like a ball behind the pubes, of about the size of a child's head, the uterus gradually relaxes, becomes somewhat softer and larger, and continues to increase by slight degrees for some hours: its maximum increase, as well as the time required to attain this size, varies considerably in different women; but we believe that we are not far off the truth in stating a little below the umbilicus as its mean height, and twenty-four hours as about the mean time it takes to attain this size, after which it begins gradually to diminish, as the works on midwifery describe, but not before. This increase in size, previous to its permanent diminution, appears to result from the state of active contraction in which the uterus was, at the moment of labour being accomplished, gradually relaxing, so that its spongy parietes are again, to a certain degree, filled with blood, although not to such an extent as to induce hemorrhage. These changes in the size of the uterus shortly after labour, although hitherto almost unnoticed, are of great importance, and form a valuable addition to our means of diagnosis.

The remaining observations under this division are of little interest. M. Hohl denies the existence of inversion of the uterus, inependent of pregnancy, as if this displacement never took place in its fullest extent with polypus. The diagnosis of inversion presents no new features beyond what we find in Richter's observations on this subject. Our author has mentioned Dewees's name once or twice in the course of the work: it is to be regretted that no reference has been made to his chapter on inversion.

The last part of Professor Hohl's work is what he calls the Special part. We cannot undertake to follow him through the whole of this: it contains a great deal of repetition and much useless digression: in fact, the greater part has been already more or less discussed, and the rest has no business here. After having gone over a wearisome description of the breasts, abdomen, pelvis, and external parts of generation, with the various anomalies which they may present, he comes to the changes which the os and cervix uteri undergo during pregnancy. We have nothing to find fault with in all this, except that the greater part has been already given. We agree with him fully where he says, "at the end of a normal pregnancy, the pains will sometimes come on for hours, or even days, before the actual commencement of labour: they go off and return without any change being observed in the os uteri. *These pains determine* the direction and position of the child." He is aware, we presume, that Fielding Ould said the same ninety-four years ago. The rest of his observations are exceedingly meagre. A few symptoms of prolapsus uteri and of inversion are mentioned; others have already occurred in another part of the work; in fact, there is no clear concise arrangement, but something of every thing, and nothing complete.

In speaking of the child's head, he gives a short and very tolerable description of what is to be felt by the finger during labour with the head in the first and second position. In these respects, he follows the views of Professor Naegelé, of Heidelberg, which, in fact, are now becoming generally received by all those who have investigated the mechanism of parturition with any degree of care. In speaking of face presentations, Professor Hohl makes a slight mistake in calling that position of the face the second where the chin is turned towards the right: this is the *first* position, and if he had considered for a moment what he had already said at page 203, (where he very correctly observes that presentations of the face are in all probability originally presentations of the head,) he would have seen why the face presents more frequently with the chin to the right than to the left. His diagnosis of a nates presentation is a right specimen of the old school. "We distinguish the breech by its rounded form, and doughy softness, by the hard tubera ischii lying near to each other, with the sulcus of the nates and the anus between them, by the parts of generation." Every assertion in this short sentence we deny. M. Hohl has, it is true, added, "the feel of the coccyx close by the edge of the anus;" and this is, in fact, the only distinguishing mark by which we can ascertain that the nates present. How are we to distinguish the round tubera ischii, the soft nates, the parts of generation, (in all probability already more or less swollen,) from the face, or even the shoulder? It is all very easy where every thing is favourable for the purpose; but, wherever the examination is in any way rendered difficult, either from the state of the os uteri, or from the presenting parts being much compressed and swollen, we can assure him that these marks will afford but an uncertain diagnosis. His description of the infant's foot from the feel which it communicates is good, and as such we present it to our readers.

"We distinguish the foot by its hardness and thickness, by the heel, by the toes, (which are short, straight, and not very moveable,) and by the ankle-joint. The foot may be distinguished from the hand by the great toe being longer than the rest, and lying close to them; whereas, the thumb is shorter than the other fingers, and stands off from these, or can be retracted. Moreover, the ends of the toes form nearly a straight line; whereas, the tips of the fingers are of unequal length. The hand is broader and softer than the foot, the fingers are longer than the toes. If the beginner be still uncertain as to what he feels, he may call to his assistance a means which may prove very useful where the membranes are not yet ruptured, and where the diagnosis may be very difficult: he has only to tickle the flat surface of the presenting part with the tip of his finger; the child will distinctly withdraw its foot: whereas, if it be the hand, this will close, or at least enable him to distinguish it by the motion of its fingers. In many cases it is impossible to know with certainty which foot it is we feel. Let us suppose the soles of the feet are turned forwards, or that we find a foot in this position: it is always that foot according to which side of the mother the great toe is directed."

There are two observations in this quotation to which we object: firstly, that the great toe of a full-grown fœtus is not always so parallel with the others as might be supposed from this description but projects from the side almost like a thumb, and in many children, shortly after birth, is capable of as great a degree of retraction; and secondly, the rule given for ascertaining which foot it is, will not hold good in practice, except in the above-mentioned case, where the toes are turned backwards. What would become of M. Hohl's diagnosis, where, as is very frequently the case during the first half of labour, the toes are either turned forwards or to one side? It surely must be far simpler to recollect that the great toe is upon the inside of the foot.

With regard to supporting the perineum, Professor Hohl fancies that he can put the head in a more convenient position than that in which it is placed by nature, and recommends a variety of manipulations to give it the necessary turn, &c. We cannot stop to offer any observations upon this subject, but merely request the obstetric reader to satisfy himself carefully as to the precise direction which the ring formed by the os externum takes upon the head at the moment of its greatest distention. He will find that in no other directions could the head offer so small a bulk; and this is with the posterior and superior quarter of the right or left parietal bone in advance, according to which position of the head it may be.

His directions for finding the orifice of the meatus urinarius for the purpose of introducing the catheter, are very objectionable: the clitoris must not be our guide on such occasions; in the first place it is a very insufficient guide, and secondly, it ought not to be touched by the finger. The best and simplest method is to pass the finger into the vaginal entrance: we feel the cushion-like mass of the urethra at the upper part of the pubic arch, and, running the finger along it, cannot fail in coming to its anterior extremity and detecting the orifice. Professor Hohl, to our surprise, advocates reposition of the umbilical cord, where it has been prolapsed, and gravely directs this and that manipulation for its reduction, according to the position of the head and the side at which the cord is prolapsed. We offer no comment on such rules for practice, but merely assure our English readers, that such is not the practice generally in Germany. His directions in turning are little better. Does M. Hohl forget what Peu, Deleurye, Hamilton, Boer, &c. have said, when he ventures to recommend rupturing the membranes as soon as the hand has passed the os uteri?

The few remaining pages on the application of the forceps, perforation, and artificial removal of the placenta, contain nothing of peculiar interest. An appendix of two pages and a half on the importance of the gustatory and olfactory organs to a practitioner, possesses little of peculiar value. The latter part of the subject is capable of much useful investigation, especially in the puerperal state and with new-born infants. It might have been made much more of with advantage.

We thus conclude our review of Professor Hohl's work. The book itself is a bad specimen of German paper and typography, the more so as most works in Germany are now got up in a very different style: in this respect Professor Kilian's is infinitely superior. Any further mention of this latter work we defer till a future opportunity. We have analysed M. Hohl's work with no little care, and we trust also impartially: it contains much valuable matter, which proves him to be a man of considerable experience and reading, and warmly devoted to his profession. But the quantity of meager, prosy, and in some parts positively objectionable, matter with which considerable portions are here and there loaded, forbid our approval of it. We regret it the more, as his name has received honourable mention by those for whose opinion we have the highest respect.

If we might presume to offer advice to Professor Hohl, and we do so with the best intentions, it would be to condense his two volumes into one of moderate size, and cut out all that extraneous and objectionable matter which we have so much complained of. The size of the work will, it is true, be diminished, but its value will be greatly increased.

## Figures and Tables

**Figure f1:**